# An Examination of *KCNE1* Mutations and Common Variants in Chronic Tinnitus

**DOI:** 10.3390/genes1010023

**Published:** 2010-04-28

**Authors:** Philipp G. Sand, Alexander Luettich, Tobias Kleinjung, Goeran Hajak, Berthold Langguth

**Affiliations:** 1 Department of Psychiatry, University of Regensburg, Universitaetsstr. 84, 93042 Regensburg, Germany; E-Mails: goeran.hajak@medbo.de (G.H.); berthold.langguth@medbo.de (B.L.); 2 Experimental and Clinical Neurosciences Graduate Program, University of Regensburg, Germany; E-Mail: alexander.luettich@stud.uni-regensburg.de (A.L.); 3 Department of Otorhinolaryngology, University of Regensburg, Franz-Josef-Strauss-Allee 11, 93053 Regensburg, Germany; E-Mail: tobias.kleinjung@klinik.uni-regensburg.de (T.K.)

**Keywords:** tinnitus, KCNE1, missense mutation, hearing disorder

## Abstract

Chronic tinnitus is a highly prevalent and often incapacitating condition frequently associated with sensorineural hearing loss. While its etiology remains incompletely understood there is a growing awareness of genetic factors that predispose to, or aggravate chronic tinnitus. Candidate genes for the disorder include *KCNE1*, a potassium channel subunit gene that has been implicated in maturation defects of central vestibular neurons, in Menière's disease, and in noise-induced hearing loss. 201 Caucasian outpatients with a diagnosis of chronic tinnitus were systematically screened for mutations in the *KCNE1* open reading frame and in the adjacent sequence by direct sequencing. Allele frequencies were determined for 46 known variants, plus two novel *KCNE1* mutations. These comprised one missense substitution (V47I) in the highly conserved region encoding the *KCNE1* transmembrane domain, and one rare variant in the gene's 3'UTR. When genotypes were grouped assuming dominance of the minor alleles, no significant genotype or compound genotype effects were observed on tinnitus severity. The newly identified V47I substitution argues in favor of an enlarged spectrum of mutations in hearing disorders. However, with regard to allele frequencies in healthy control populations from earlier studies, more common *KCNE1* variants are unlikely to play a major role in chronic tinnitus. Further investigations are invited to address variation in additional channel subunits as possible risk factors in tinnitus.

## 1. Introduction

Tinnitus refers to a sensation of sound perceived in the head or in the ears without any evident external stimulus. The condition may cause significant discomfort and may interfere with daily activities, emotional state and sleep. Depending on the specifications used in self-assessments of tinnitus, estimates of prevalence in the general population vary from 3% to 30% in epidemiological studies [1,2]. To date, the etiology remains largely unknown but an established association with various forms of sensorineural hearing impairment and frequent precipitation by noise exposure suggest substantial overlap with pathologies of the inner ear [3], and related disorders of auditory information processing [4]. In contrast to well-defined environmental risk factors, however, only limited data are currently available on genetic traits that may predispose to common, chronic forms of tinnitus (reviewed by [5]). 

Voltage-gated ion channels that directly control the neural transmission of auditory input are strong candidates for the pathophysiology of tinnitus. In the inner ear, sensory neurons are surrounded by endolymph rich in KCl and constantly recycle potassium for the generation of endocochlear potentials [6]. K^+^ homeostasis requires coexpression of α and β subunits of pore-forming channel proteins in the lateral wall of the cochlea and the vestibular labyrinth [7]. Dysfunctional channels and mutations in the gene encoding the KCNQ4 subunit are a hallmark of autosomal dominant deafness 2A [8]. Mutated KCNQ1 α and KCNE1 β subunits, in turn, cause syndromal deafness with abnormal cardiac ventricular repolarization (Jervell and Lange-Nielsen Syndrome, JLNS) [9,10,11]. A prominent role of the KCNE1 subunit in auditory perception is underscored by degeneration of sensory hair cells and deafness in *KCNE1* knock-out animals [12], plus deleterious effects of a spontaneous KCNE1 null mutation on hearing in mice [13]. In addition, KCNE1 regulates trafficking and activation of another potassium channel, KCNH3, in the cerebral cortex and in other parts of the brain that have been implicated in disorders of excitability and synchronization [14].

Common genetic variation in potassium channel genes has recently been proposed as a possible risk modifier in Menière's disease [15], in age-related hearing loss [16], and in noise-induced hearing loss [17,18], *i.e.,* in conditions that typically co-occur with tinnitus [19]. We hypothesized that primary chronic tinnitus could be part of the phenotypic spectrum associated with *KCNE1*, and systematically screened the open reading frame for variants in subjects who had experienced tinnitus for a minimum of six months.

## 2. Results and Discussion

**Figure 1:**
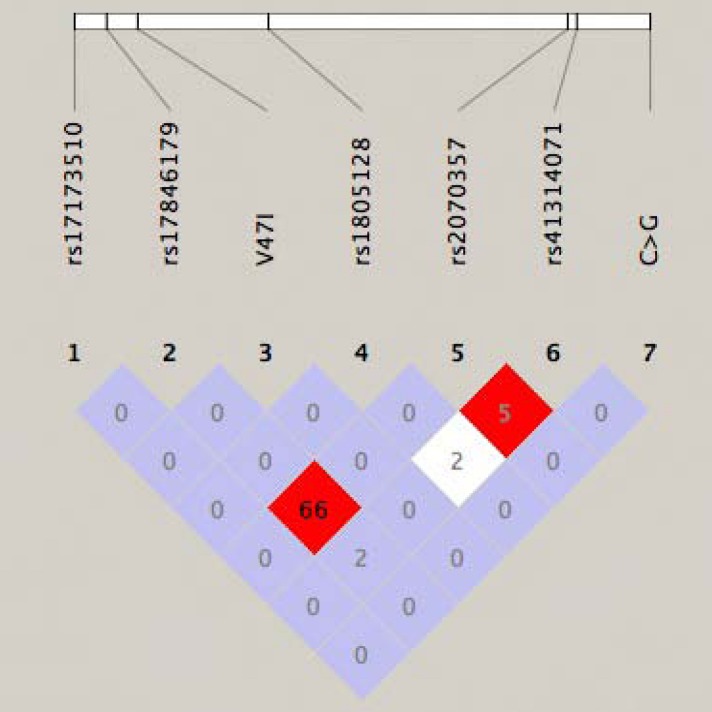
LD plot and R^2^ values for the seven *KCNE1* variants identified.

**Figure 2:**
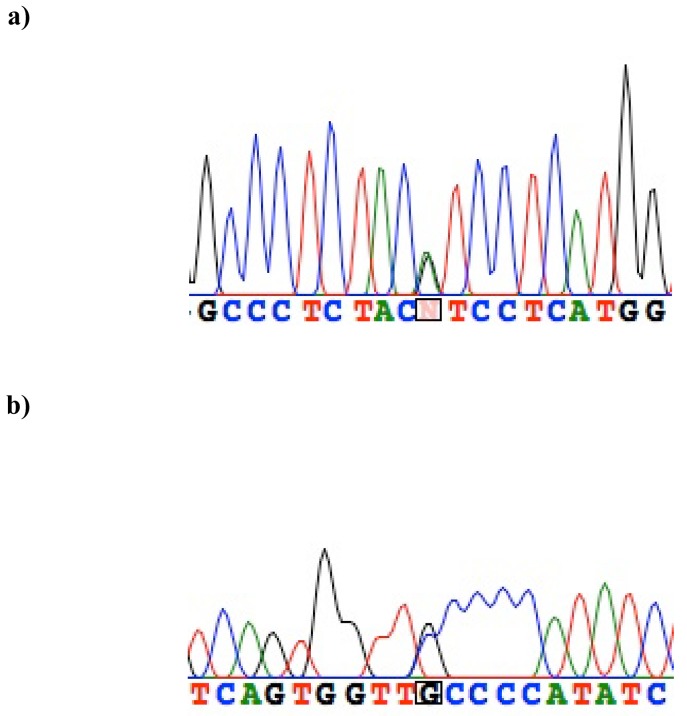
Chromatograms of the newly identified *KCNE1* Val47Ile **(a)** and noncoding C>G substitution in the 3'UTR **(b)**.

**Figure 3:**
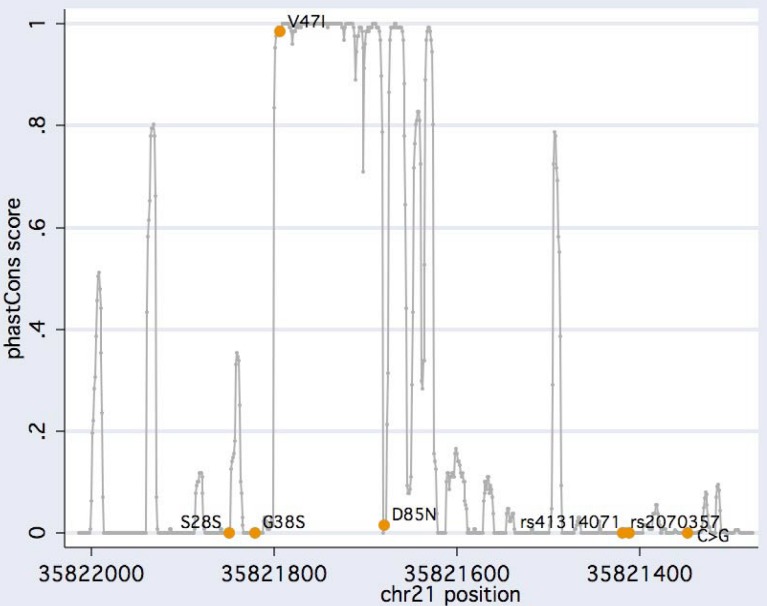
Comparative analysis of the *KCNE1* genomic sequence screened. Basewise conservation scores obtained with the Multiz alignment are plotted against the physical position on chromosome 21 for 31 placental mammals featured in the UCSC Genome Browser. The newly identified V47I mutation maps to the highly conserved *KCNE1* transmembrane domain delimited by residues 44 and 60 [61].

We identified four coding and three noncoding variants with minor allele frequencies ranging from 0.002 to 0.45 (Table 1, Figure 1). These included one silent polymorphism, S28S, two known missense substitutions, S38G and D85N, plus a novel missense variant, V47I (Figure 2a). This newly identified substitution maps to a highly conserved region encoding the *KCNE1* transmembrane domain (TMD) (Figure 3). Of the SNPs located in the 3' untranslated region, two had been previously described (rs2070357 and rs41314071) and one was a novel transversion occurring in <1% of alleles (Figure 2b). Three common haplotypes were defined by markers rs17846179 (S38G) and rs2070357: f_GG_ = 0.544, f_AA_ = 0.358, and f_AG_ = 0.097. 

**Table 1:** Observed allele frequencies for the *KCNE1* sequence screened in subjects with chronic tinnitus (N=201). Seven non-monomorphic variants are shaded.

dbSNP ID	chr21 position	major>minor alleles^a^	variant amino acid	minor allele frequency in chronic tinnitus	homozygous/heterozygous carriers of the minor allele (p_HWE_)
rs28933384	35,821,913	C>T	T7I	0.000	-
-	35,821,910	C>T	A8V	0.000	-
-	35,821,904	C>T	T10M	0.000	-
-	35,821,903	G>A	T10T	0.000	-
-	35,821,883	G>A	W17X	0.000	-
-	35,821,874	C>T	T20I	0.000	-
-	35,821,850	C>T	S28L	0.000	-
rs17173510	35,821,849	G>A	S28S	0.002	0/1 (0.972)
rs17857111	35,821,838	G>A	R32H	0.000	-
-	35,821,826	G>A	R36H	0.000	-
rs1805127	35,821,821	G>A	G38S	0.359	28/88 (0.498)
-	35,821,794	G>T	V47F	0.000	-
(novel)	35,821,794	G>A	V47I	0.002	0/1 (0.972)
-	35,821,780-1	TG>AC	L51H	0.000	-
rs17173509	35,821,778	G>C	G52A	0.000	-
-	35,821,779	G>A	G52R	0.000	-
-	35,821,775	T>C	F53S	0.000	-
-	35,821,774	C>T	F53F	0.000	-
rs17173508	35,821,771	C>T	F54F	0.000	-
-	35,821,770	G>A	G55S	0.000	-
-	35,821,761	A>C	T58P	0.000	-
dbSNP ID	chr21 position	major>minor alleles^a^	variant amino acid	minor allele frequency in chronic tinnitus	homozygous/heterozygous carriers of the minor allele (p_HWE_)
-	35,821,757	T>C	L59P	0.000	-
-	35,821,734	C>T	R67C	0.000	-
-	35,821,733	G>A	R67H	0.000	-
-	35,821,727	A>G	K69R	0.000	-
-	35,821,724	A>T	K70M	0.000	-
-	35,821,723	G>C	K70N	0.000	-
-	35,821,712	C>T	S74L	0.000	-
-	35,821,708	C>T	N75N	0.000	-
-	35,821,707	G>A	D76N	0.000	-
-	35,821,693	C>G	V80V	0.000	-
-	35,821,693	C>T	V80V	0.000	-
-	35,821,691	A>G	Y81C	0.000	-
-	35,821,686	G>A	E83K	0.000	-
rs1805128	35,821,680	G>A	D85N	0.007	0/3 (0.915)
-	35,821,674	T>C	W87R	0.000	-
-	35,821,641	C>T	R98W	0.000	-
rs17853625	35,821,615	C>A	C106X	0.000	-
-	35,821,608	G>A	V109I	0.000	-
-	35,821,584	C>T	Q117X	0.000	-
-	35,821,559	C>T	T125M	0.000	-
-	35,821,554	C>A	P127T	0.000	-
rs2070357	35,821,419	G>A	-	0.455	42/98 (0.865)
rs41314071	35,821,411	A>G	-	0.045	1/16 (0.328)
rs41314069	35,821,376	C>A	-	0.000	-
(novel)	35,821,347	C>G	-	0.003	0/1 (0.972)
rs41312371	35,821,283	A>C	-	0.000	-
rs41314807	35,821,275	C>T	-	0.000	-

^a^ all alleles refer to the chr21 minus strand

For all coding variants identified, reference allele frequencies in healthy Caucasian populations were obtained from the literature and from variation databases. Data from a Polish control population [26] were incongruent with all remaining studies and considered to be misleading. When allele frequencies in the other control populations were compared to the respective frequencies in tinnitus subjects assuming all controls were tinnitus-free, no significant difference was noted for S28S, G38S, V47I, and D85N (Table 2). For non-coding variants, a comparison of allele frequencies in tinnitus patients with reference frequencies retrieved from dbSNP [27] (HapMap CEU, N=59, rs2070357, plus the Coriell Cell Repository Caucasian panel, N=47, rs2070357 and rs41314071) with the Genome Variation Server [28] gave non-significant association results (data not shown). Based on the entire sample of tinnitus patients and nine Caucasian control populations, however, a weak effect on the susceptibility to tinnitus cannot be entirely ruled out. Thus power simulations indicated that we should require over 12,500 patients in order to exclude a modifying role of G38S on allelic risk with a statistical power of 0.8.

**Table 2:** Reference frequencies of *KCNE1* coding variants in Caucasians as reported for unrelated, healthy controls. Of these, five control populations ([36,52,58,59,60], total N=938) have been systematically screened for mutations and serve as a reference for the novel V47I variant. One further study involving 100 Canadian controls [31] was excluded as allele frequencies were missing. Data reported by Prystupa *et al.*[26] are given in brackets to indicate a likely misallocation of major and minor alleles. When this figure is excluded, exact tests of allelic association conducted with reference populations and the tinnitus sample give non-significant (n.s.) results throughout.

healthy controls (N_unrelated_)	source	f_Ser28(TCA)_	*vs.* f_Ser28(TCA)_ in present study (*p*)	f_Ser38_	*vs.* f_Ser38_ in present study (*p*)	f_Ile47_	*vs.* f_Ile47_ in present study (*p*)	f_Asn85_	*vs.* f_Asn85_ in present study (*p*)
U.S., European descent (187)	[36]	0.000	n.s.	-	-	0.000	n.s.	-	-
Dutch (32)	[58]	0.000	n.s.	0.33	n.s.	0.000	n.s.	0.000	n.s.
German (141)	[59]	-	-	-	-	0.000	n.s.	-	-
French (398)	[60,62]	0.000	n.s.	0.372	n.s.	0.000	n.s.	0.018	n.s.
Polish (129)	[26]	-	-	(0.582)	(<0.0001)	-	-	-	-
German (3,916)	[63]	-	-	0.368	n.s.	-	-	-	-
Finnish (5,043)	[64]	-	-	-	-	-	-	0.014	n.s.
U.S., European descent (180)	[51]	0.006	n.s.	0.378	n.s.	0.000	n.s.	0.008	n.s.
Central Europeans (59)	[27] HapMap CEU	-	-	0.381	n.s.	-	-	0.008	n.s.
Caucasian panel (47)	[27] Coriell Cell Repository R31 CAU	-	-	0.394	n.s.	-	-	0.021	n.s.

**Figure 4:**
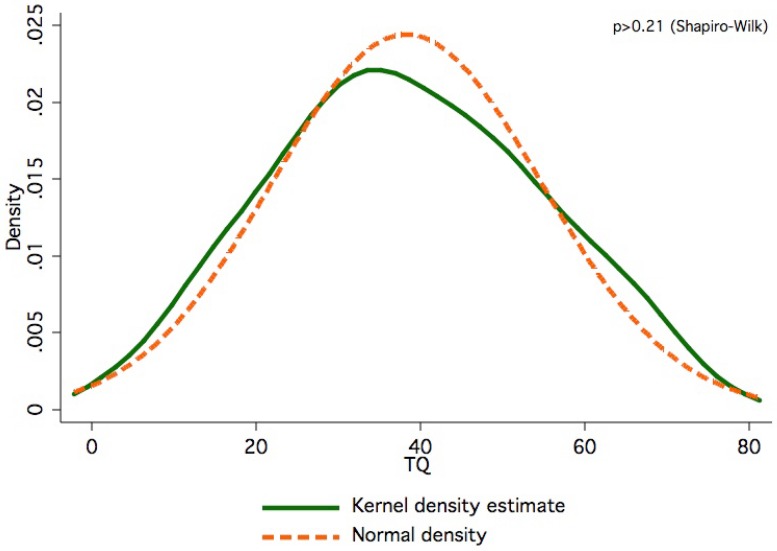
Distribution of TQ scores in 183 subjects with chronic tinnitus.

With regard to the severity of symptoms, TQ scores followed a Gaussian distribution (Figure 4) and averaged 38.3 ±16.3 (mean ±SD) out of 84 points (N=183). By this measure, tinnitus was rated 'mild' (0 to 30 points) in 34.4%, 'moderate' (31 to 46 points) in 33.9%, 'severe' (47 to 59 points) in 20.2%, and 'extreme' (60 to 84 points) in 11.5% of subjects investigated. Carrier of the V47I substitution was a 66 year-old woman who self-graded her tinnitus as 'extreme', scoring 62 out of 84 points on the TQ scale, and above the 91^st^ percentile. She had suffered from tinnitus for 4.5 years but did not present with hearing impairment. In three individuals heterozygous for the D85N substitution (f=0.007), tinnitus severity was rated 'moderate' (36, 38 and 44 points). Neither V47I nor any other genotype or compound genotype predicted tinnitus severity regardless of concomitant hearing impairment (ANOVA, F=0.89, df=7, p>0.51).

Eight additional *KCNE1* variants listed in dbSNP were not confirmed in our sample (rs28933384, rs17857111, rs17173509, rs17173508, rs17853625, rs41314069, rs41312371, and rs41314807). A number of previously reported KCNE1 mutations were also excluded: A8V [29,30], T10M [30], T10T [31], W17X [30], T20I [32], S28L [30,33], R36H [34], V47F [35], L51H [35], G52A [36], G52R [37], F53S [34], G55S [30], T58P [11,30], L59P [11,30], R67C [30], R67H [30], K69R [36], K70M [30], K70N [38], S74L [39,40], N75N [41], D76N [30,39,41,42,43,44,45], V80V [46,47], Y81C [38,48], E83K [30], W87R [35], R98W [29,39,49], V109I [36,50], Q117X [30], T125M [30], and P127T [40,51] (Table 1).

Allele frequencies of known coding variants compare to data previously reported in healthy Caucasian populations with one exception [26]. In the latter study, inverted allele counts suggest a misallocation of G38 and S38 (Table 2). The present lack of significant differences in *KCNE1* coding allele frequencies of control subjects and tinnitus patients is thus based on a comparison with nine populations previously investigated. While this approach was not adequately powered to rule out a causative role of gene variants in tinnitus, our results tend to further disprove *KCNE1* variation as a risk factor in pathologies with complex modes of inheritance. For Menière's disease, Doi *et al.* [15] had originally claimed an association with S38 in an Asian population but recent work has cast doubt on the validity of these findings by exposing stratification artefacts and by providing independent negative association results for both G38S and D85N [52]. With regard to noise-induced hearing loss, a risk-enhancing effect of the G38 allele disappeared after correcting for multiple testing [17]. A recent attempt to corroborate this weak association in a separate Caucasian sample was unsuccessful [18]. The D85 allele, in turn, has been labeled both 'risk-enhancing' and 'protective' in noise-induced hearing loss [17,18]. Others have classified the substitution as an infrequent polymorphism rather than a disease-causing mutation [36,53,54].

To date, *in vitro* analyses have failed to resolve the controversies surrounding a putative *in vivo* impact of G38S and D85N on potassium conductance. The G38S substitution did not show any major effects on KCNE1 glycolsylation [55] but has not been examined in a heterologous expression system. Patch-clamp experiments with Chinese hamster ovarian cells and *Xenopus laevis* oocytes expressing D85N have yielded contradictory effects on opening of the potassium channel, *i.e.,* a gain of function [17] and a loss of function [40,56]. While the newly identified V47I mutation awaits further characterization in expression models, an earlier study has addressed a compound heterozygous TMD substitution involving the same residue (V47F + L51H) in a case of mild JLNS [35]. Coexpression of V47F + L51H mutant cRNA in *Xenopus* oocytes gave KCNQ1 activation currents indistinguishable from those elicited by simple *KCNE1* V47F mutants, and led the authors to assume that the phenotype was primarily caused by functional effects of V47F.

*KCNE1* TMD missense mutations have been described in cases of JLNS (V47F, L51H, T58P, and L59P), long QT syndrome (G52R, F53S, and G55S), and on one occasion, in an anonymous subject classified as 'apparently healthy' (G52A). To judge by the non-identification of V47I in systematic screenings of healthy controls, V47I is rare in the Caucasian general population (Table 2) and has not been observed either in African American or in Asian control populations [36,38]. Pending further characterization of V47I effects on KCNE1 function *in vitro*, a causative role in tinnitus etiology remains speculative. Extreme symptom severity in the mutation carrier would appear to strengthen the genotype-phenotype relationship but family data and additional data on cardiac repolarization were unavailable. In analogy to the established comorbidity of hearing disorders and arryhthmias in JLNS, the spectrum of monogenic disorders associated with *KCNE1* mutations may involve rare cases of tinnitus. It is noteworthy that a 'cardiac irregularity' is also mentioned as an accessory symptom in one of the earliest scientific accounts of tinnitus [57].

## 3. Experimental Section

In 201 German outpatients (152 men and 49 women, age 49.9 ±12.0 yrs, mean ±SD) consulting for chronic tinnitus, the diagnosis was confirmed by a detailed neurootological examination including otoscopy, stapedius reflexes, middle ear pressure measurements and pure tone audiometry. For the present study, only patients with subjective tinnitus were included. Tinnitus severity was assessed by the Tinnitus Questionnnaire (TQ) [20] in 183 patients (90.6%).

Genomic DNA was extracted from lymphocytes using standard procedures prior to amplification of the *KCNE1* coding region by PCR. Briefly, a 765bp amplicon was generated using the following oligomers: 5'-TTT TGA TTT GGG GTT GCA T-3' (forward) and 5'-GCT AGC TGC AAG GGA GTC T-3' (reverse). PCR products were purified with ExoSAP-IT (GE Healthcare, Freiburg, Germany) for custom sequencing and for the identification of DNA variants by comparison with the human genome reference (Genome Reference Consortium Build 37, February 2009 release). Multiple sequence alignments were conducted with DNA Dynamo 1.0 (Blue Tractor Software, UK). STATA 8.0 (Stata Corporation, College Station, TX, USA) was used for descriptive statistics, for conducting tests of allelic association, and for modeling effects of *KCNE1* genotypes on TQ scores by ANOVA. To this avail, genotypes were dichotomized using a dominant model for minor alleles. *KCNE1* allele frequencies from reference populations were compared to the present data using Fisher's exact test. The Shapiro-Wilk statistic served to test the null hypothesis of normally distributed TQ scores. The level of statistical significance was set at *p*=0.05. All *p* values are uncorrected for multiple testing. 

For estimating the functionality of sequence variants observed in our sample, evolutionary conservation was assessed with a phylogenetic hidden Markov model-based method, phastCons, that describes the process of DNA substitution at each site in a genome and the way this process changes from one site to the next [21]. Genomic sequences from 31 placental mammals were aligned to the human reference delimited by forward and reverse primers using a Threaded Blockset Aligner [22] as implemented in the conservation track of the UCSC Genome Browser [23]. Power simulations were conducted with PS 1.0.15 [24]. Linkage disequilibrium and conformity of genotype distributions with the Hardy-Weinberg equilibrium was measured with HaploView 4.2 [25]. 

## 4. Conclusions

Taken together, the present findings lend little support to the notion of common *KCNE1* variants as possible risk modifiers of chronic tinnitus, but suggest the existence of syndromal subtypes with underlying channelopathies and invite more detailed investigations of other genes relevant to potassium homeostasis. Should such tinnitus channelopathies be confirmed in the future, the existing options for prevention and treatment of the disorder will need to be reappraised.

## References

[B1-genes-01-00023] Sanchez L. (2004). The epidemiology of tinnitus. Audiol. Med..

[B2-genes-01-00023] Hoffman H.J., Reed G.W. (2004). Epidemiology of Tinnitus. Tinnitus: Theory and Management.

[B3-genes-01-00023] Eggermont J.J. (2007). Pathophysiology of tinnitus. Prog. Brain Res..

[B4-genes-01-00023] Attias J., Furman V., Shemesh Z., Bresloff I. (1996). Impaired brain processing in noise-induced tinnitus patients as measured by auditory and visual event-related potentials. Ear Hear..

[B5-genes-01-00023] Sand P.G., Langguth B., Kleinjung T., Eichhammer P. (2007). Genetics of chronic tinnitus. Prog. Brain Res..

[B6-genes-01-00023] Wangemann P. (2002). K+ cycling and the endocochlear potential. Hear Res..

[B7-genes-01-00023] Hibino H., Nin F., Tsuzuki C., Kurachi Y. (2010). How is the highly positive endocochlear potential formed? The specific architecture of the stria vascularis and the roles of the ion-transport apparatus. Pflugers Arch..

[B8-genes-01-00023] Coucke P.J., Van Hauwe P., Kelley P.M., Kunst H., Schatteman I., Van Velzen D., Meyers J., Ensink R.J., Verstreken M., Declau F., Marres H., Kastury K., Bhasin S., McGuirt W.T., Smith R.J.H., Cremers C.W.R.J., Van de Heyning P., Willems P.J., Smith S.D., Van Camp G. (1999). Mutations in the KCNQ4 gene are responsible for autosomal dominant deafness in four DFNA2 families. Hum. Molec. Genet..

[B9-genes-01-00023] Jervell A., Lange-Nielsen F. (1957). Congenital deaf-mutism, functional heart disease with prolongation of Q-T interval and sudden death. Am. Heart J..

[B10-genes-01-00023] Neyroud N., Tesson F., Denjoy I., Leibovici M., Donger C., Barhanin J., Faure S., Gary F., Coumel P., Petit C., Schwartz K., Guicheney P. (1997). A novel mutation in the potassium channel gene KVLQT1 causes the Jervell and Lange-Nielsen cardioauditory syndrome. Nature Genet..

[B11-genes-01-00023] Tyson J., Tranebjaerg L., Bellman S., Wren C., Taylor J.F., Bathen J., Aslaksen B., Sørland S.J., Lund O., Malcolm S., Pembrey M., Bhattacharya S., Bitner-Glindzicz M. (1997). IsK and KvLQT1: mutation in either of the two subunits of the slow component of the delayed rectifier potassium channel can cause Jervell and Lange-Nielsen syndrome. Hum. Mol. Genet..

[B12-genes-01-00023] Vetter D.E., Mann J.R., Wangemann P., Liu J., McLaughlin K.J., Lesage F., Marcus D.C., Lazdunski M., Heinemann S.F., Barhanin J. (1996). Inner ear defects induced by null mutation of the isk gene. Neuron.

[B13-genes-01-00023] Letts V.A., Valenzuela A., Dunbar C., Zheng Q.Y., Johnson K.R., Frankel W.N. (2000). A new spontaneous mouse mutation in the Kcne1 gene. Mamm. Genome.

[B14-genes-01-00023] Clancy S.M., Chen B., Bertaso F., Mamet J., Jegla T. (2009). KCNE1 and KCNE3 beta-subunits regulate membrane surface expression of Kv12.2 K(+) channels *in vitro* and form a tripartite complex *in vivo*. PLoS One.

[B15-genes-01-00023] Doi K., Sato T., Kuramasu T., Hibino H., Kitahara T., Horii A., Matsushiro N., Fuse Y., Kubo T. (2005). Ménière’s disease is associated with single nucleotide polymorphisms in the human potassium channel genes, KCNE1 and KCNE3. ORL J. Otorhinolaryngol. Relat. Spec..

[B16-genes-01-00023] Van Eyken E., Van Laer L., Fransen E., Topsakal V., Lemkens N., Laureys W., Nelissen N., Vandevelde A., Wienker T., Van De Heyning P., Van Camp G. (2006). KCNQ4: a gene for age-related hearing impairment?. Hum. Mutat..

[B17-genes-01-00023] Van Laer L., Carlsson P.I., Ottschytsch N., Bondeson M.L., Konings A., Vandevelde A., Dieltjens N., Fransen E., Snyders D., Borg E., Raes A., Van Camp G. (2006). The contribution of genes involved in potassium-recycling in the inner ear to noise-induced hearing loss. Hum. Mutat..

[B18-genes-01-00023] Pawelczyk M., Van Laer L., Fransen E., Rajkowska E., Konings A., Carlsson P.I., Borg E., Van Camp G., Sliwinska-Kowalska M. (2009). Analysis of gene polymorphisms associated with K ion circulation in the inner ear of patients susceptible and resistant to noise-induced hearing loss. Ann. Hum. Genet..

[B19-genes-01-00023] Axelsson A., Sandh A. (1985). Tinnitus in noise-induced hearing loss. Br. J. Audiol..

[B20-genes-01-00023] Goebel G., Hiller W. (1994). The tinnitus questionnaire. A standard instrument for grading the degree of tinnitus. Results of a multicenter study with the tinnitus questionnaire. HNO.

[B21-genes-01-00023] Siepel A., Bejerano G., Pedersen J.S., Hinrichs A.S., Hou M., Rosenbloom K., Clawson H., Spieth J., Hillier L.W., Richards S., Weinstock G.M., Wilson R.K., Gibbs R.A., Kent W.J., Miller W., Haussler D. (2005). Evolutionarily conserved elements in vertebrate, insect, worm, and yeast genomes. Genome Res..

[B22-genes-01-00023] Blanchette M., Kent W.J., Riemer C., Elnitski L., Smit A.F., Roskin K.M., Baertsch R., Rosenbloom K., Clawson H., Green E.D., Haussler D., Miller W. (2004). Aligning multiple genomic sequences with the threaded blockset aligner. Genome Res..

[B23-genes-01-00023] Rhead B., Karolchik D., Kuhn R.M., Hinrichs A.S., Zweig A.S., Fujita P.A., Diekhans M., Smith K.E., Rosenbloom K.R., Raney B.J., Pohl A., Pheasant M., Meyer L.R., Learned K., Hsu F., Hillman-Jackson J., Harte R.A., Giardine B., Dreszer T.R., Clawson H., Barber G.P., Haussler D., Kent W.J. (2010). The UCSC Genome Browser database: update 2010. Nucleic Acids Res..

[B24-genes-01-00023] Dupont W.D., Plummer Jr W.D.. (1990). Power and sample size calculations: a review and computer program. Control Clin. Trials.

[B25-genes-01-00023] Barrett J.C., Fry B., Maller J., Daly M.J. (2005). Haploview: analysis and visualization of LD and haplotype maps. Bioinformatics.

[B26-genes-01-00023] Prystupa A., Dzida G., Myśliński W., Małaj G., Lorenc T. (2006). MinK gene polymorphism in the pathogenesis of lone atrial fibrillation. Kardiol. Pol..

[B27-genes-01-00023] Sayers E.W., Barrett T., Benson D.A., Bolton E., Bryant S.H., Canese K., Chetvernin V., Church D.M., Dicuccio M., Federhen S., Feolo M., Geer L.Y., Helmberg W., Kapustin Y., Landsman D., Lipman D.J., Lu Z., Madden T.L., Madej T., Maglott D.R., Marchler-Bauer A., Miller V., Mizrachi I., Ostell J., Panchenko A., Pruitt K.D., Schuler G.D., Sequeira E., Sherry S.T., Shumway M., Sirotkin K., Slotta D., Souvorov A., Starchenko G., Tatusova T.A., Wagner L., Wang Y., John Wilbur W., Yaschenko E., Ye J. (2010). Database resources of the National Center for Biotechnology Information. Nucleic Acids Res..

[B28-genes-01-00023] Genome Variation Server. http://gvs.gs.washington.edu/GVS.

[B29-genes-01-00023] Ohno S., Zankov D.P., Yoshida H., Tsuji K., Makiyama T., Itoh H., Akao M., Hancox J.C., Kita T., Horie M. (2007). N- and C-terminal KCNE1 mutations cause distinct phenotypes of long QT syndrome. Heart Rhythm.

[B30-genes-01-00023] Kapplinger J.D., Tester D.J., Salisbury B.A., Carr J.L., Harris-Kerr C., Pollevick G.D., Wilde A.A., Ackerman M.J. (2009). Spectrum and prevalence of mutations from the first 2,500 consecutive unrelated patients referred for the FAMILION long QT syndrome genetic test. Heart Rhythm.

[B31-genes-01-00023] Koo S.H., Ho W.F., Lee E.J. (2006). Genetic polymorphisms in KCNQ1, HERG, KCNE1 and KCNE2 genes in the Chinese, Malay and Indian populations of Singapore. Br. J. Clin. Pharmacol..

[B32-genes-01-00023] Millat G., Kugener B., Chevalier P., Chahine M., Huang H., Malicier D., Rodriguez-Lafrasse C., Rousson R. (2009). Contribution of long-QT syndrome genetic variants in sudden infant death syndrome. Pediatr. Cardiol..

[B33-genes-01-00023] Shim S.H., Ito M., Maher T., Milunsky A. (2005). Gene sequencing in neonates and infants with the long QT syndrome. Genet. Test.

[B34-genes-01-00023] Napolitano C., Priori S.G., Schwartz P.J., Bloise R., Ronchetti E., Nastoli J., Bottelli G., Cerrone M., Leonardi S. (2005). Genetic testing in the long QT syndrome: development and validation of an efficient approach to genotyping in clinical practice. J.A.M.A..

[B35-genes-01-00023] Bianchi L., Shen Z., Dennis A.T., Priori S.G., Napolitano C., Ronchetti E., Bryskin R., Schwartz P.J., Brown A.M. (1999). Cellular dysfunction of LQT5-minK mutants: abnormalities of IKs, IKr and trafficking in long QT syndrome. Hum. Mol. Genet..

[B36-genes-01-00023] Ackerman M.J., Tester D.J., Jones G.S., Will M.L., Burrow C.R., Curran M.E. (2003). Ethnic differences in cardiac potassium channel variants: implications for genetic susceptibility to sudden cardiac death and genetic testing for congenital long QT syndrome. Mayo Clin. Proc..

[B37-genes-01-00023] Ma L., Lin C., Teng S., Chai Y., Bähring R., Vardanyan V., Li L., Pongs O., Hui R. (2003). Characterization of a novel Long QT syndrome mutation G52R-KCNE1 in a Chinese family. Cardiovasc. Res..

[B38-genes-01-00023] Lai L.P., Su Y.N., Hsieh F.J., Chiang F.T., Juang J.M., Liu Y.B., Ho Y.L., Chen W.J., Yeh S.J., Wang C.C., Ko Y.L., Wu T.J., Ueng K.C., Lei M.H., Tsao H.M., Chen S.A., Lin T.K., Wu M.H., Lo H.M., Huang S.K., Lin J.L. (2005). Denaturing high-performance liquid chromatography screening of the long QT syndrome-related cardiac sodium and potassium channel genes and identification of novel mutations and single nucleotide polymorphisms. J. Hum. Genet..

[B39-genes-01-00023] Splawski I., Tristani-Firouzi M., Lehmann M.H., Sanguinetti M.C., Keating M.T. (1997). Mutations in the hminK gene cause long QT syndrome and suppress IKs function. Nat. Genet..

[B40-genes-01-00023] Westenskow P., Splawski I., Timothy K.W., Keating M.T., Sanguinetti M.C. (2004). Compound mutations: a common cause of severe long-QT syndrome. Circulation.

[B41-genes-01-00023] Berge K.E., Haugaa K.H., Früh A., Anfinsen O.G., Gjesdal K., Siem G., Oyen N., Greve G., Carlsson A., Rognum T.O., Hallerud M., Kongsgård E., Amlie J.P., Leren T.P. (2008). Molecular genetic analysis of long QT syndrome in Norway indicating a high prevalence of heterozygous mutation carriers. Scand. J. Clin. Lab. Invest..

[B42-genes-01-00023] Schulze-Bahr E., Wang Q., Wedekind H., Haverkamp W., Chen Q., Sun Y., Rubie C., Hördt M., Towbin J.A., Borggrefe M., Assmann G., Qu X., Somberg J.C., Breithardt G., Oberti C., Funke H. (1997). KCNE1 mutations cause jervell and Lange-Nielsen syndrome. Nat. Genet..

[B43-genes-01-00023] Duggal P., Vesely M.R., Wattanasirichaigoon D., Villafane J., Kaushik V., Beggs A.H. (1998). Mutation of the gene for IsK associated with both Jervell and Lange-Nielsen and Romano-Ward forms of Long-QT syndrome. Circulation.

[B44-genes-01-00023] Tester D.J., Will M.L., Haglund C.M., Ackerman M.J. (2005). Compendium of cardiac channel mutations in 541 consecutive unrelated patients referred for long QT syndrome genetic testing. Heart Rhythm.

[B45-genes-01-00023] Tester D.J., Arya P., Will M., Haglund C.M., Farley A.L., Makielski J.C., Ackerman M.J. (2006). Genotypic heterogeneity and phenotypic mimicry among unrelated patients referred for catecholaminergic polymorphic ventricular tachycardia genetic testing. Heart Rhythm.

[B46-genes-01-00023] Friedlander Y., Vatta M., Sotoodehnia N., Sinnreich R., Li H., Manor O., Towbin J.A., Siscovick D.S., Kark J.D. (2005). Possible association of the human KCNE1 (minK) gene and QT interval in healthy subjects: evidence from association and linkage analyses in Israeli families. Ann. Hum. Genet..

[B47-genes-01-00023] Ellinor P.T., Petrov-Kondratov V.I., Zakharova E., Nam E.G., MacRae C.A. (2006). Potassium channel gene mutations rarely cause atrial fibrillation. BMC Med. Genet..

[B48-genes-01-00023] Wu D.M., Lai L.P., Zhang M., Wang H.L., Jiang M., Liu X.S., Tseng G.N. (2006). Characterization of an LQT5-related mutation in KCNE1, Y81C: implications for a role of KCNE1 cytoplasmic domain in IKs channel function. Heart Rhythm.

[B49-genes-01-00023] Millat G., Chevalier P., Restier-Miron L., Da Costa A., Bouvagnet P., Kugener B., Fayol L., Gonzàlez Armengod C., Oddou B., Chanavat V., Froidefond E., Perraudin R., Rousson R., Rodriguez-Lafrasse C. (2006). Spectrum of pathogenic mutations and associated polymorphisms in a cohort of 44 unrelated patients with long QT syndrome. Clin. Genet..

[B50-genes-01-00023] Schulze-Bahr E., Schwarz M., Hauenschild S., Wedekind H., Funke H., Haverkamp W., Breithardt G., Pongs O., Isbrandt D. (2001). A novel long-QT 5 gene mutation in the C-terminus (V109I) is associated with a mild phenotype. J. Mol. Med..

[B51-genes-01-00023] Splawski I., Shen J., Timothy K.W., Lehmann M.H., Priori S., Robinson J.L., Moss A.J., Schwartz P.J., Towbin J.A., Vincent G.M., Keating M.T. (2000). Spectrum of mutations in long-QT syndrome genes. KVLQT1, HERG, SCN5A, KCNE1, and KCNE2. Circulation.

[B52-genes-01-00023] Campbell C.A., Della Santina C.C., Meyer N.C., Smith N.B., Myrie O.A., Stone E.M., Fukushima K., Califano J., Carey J.P., Hansen M.R., Gantz B.J., Minor L.B., Smith R.J. (2010). Polymorphisms in KCNE1 or KCNE3 are not associated with Ménière disease in the Caucasian population. Am. J. Med. Genet. A..

[B53-genes-01-00023] Tesson F., Donger C., Denjoy I., Berthet M., Bennaceur M., Petit C., Coumel P., Schwarts K., Guicheney P. (1996). Exclusion of KCNE1 (IsK) as a candidate gene for Jervell and Lange-Nielsen syndrome. J. Mol. Cell. Cardiol..

[B54-genes-01-00023] Jongbloed R., Marcelis C., Velter C., Doevendans P., Geraedts J., Smeets H. (2002). DHPLC analysis of potassium ion channel genes in congenital long QT syndrome. Hum. Mutat..

[B55-genes-01-00023] Herlyn H., Zechner U., Oswald F., Pfeufer A., Zischler H., Haaf T. (2009). Positive selection at codon 38 of the human KCNE1 (= minK) gene and sporadic absence of 38Ser-coding mRNAs in Gly38Ser heterozygotes. BMC Evol. Biol..

[B56-genes-01-00023] Nishio Y., Makiyama T., Itoh H., Sakaguchi T., Ohno S., Gong Y.Z., Yamamoto S., Ozawa T., Ding W.G, Kawamura M., Akao M., Matsuura H., Kimura T., Kita T., Horie M. (2009). D85N, a KCNE1 polymorphism, is a disease-causing gene variant in long QT syndrome. J. Am. Coll. Cardiol..

[B57-genes-01-00023] Jones M. (1890). A discussion on the etiology of tinnitus aurium. Br. Med. J..

[B58-genes-01-00023] Paulussen A.D., Gilissen R.A., Armstrong M., Doevendans P.A., Verhasselt P., Smeets H.J., Schulze-Bahr E., Haverkamp W., Breithardt G., Cohen N., Aerssens J. (2004). Genetic variations of KCNQ1, KCNH2, SCN5A, KCNE1, and KCNE2 in drug-induced long QT syndrome patients. J. Mol. Med..

[B59-genes-01-00023] Aydin A., Bähring S., Dahm S., Guenther U.P., Uhlmann R., Busjahn A., Luft F.C. (2005). Single nucleotide polymorphism map of five long-QT genes. J. Mol. Med..

[B60-genes-01-00023] Gouas L., Nicaud V., Chaouch S., Berthet M., Forhan A., Tichet J., Tiret L., Balkau B., Guicheney P. (2007). Confirmation of associations between ion channel gene SNPs and QTc interval duration in healthy subjects. Eur. J. Hum. Genet..

[B61-genes-01-00023] Tian C., Vanoye C.G., Kang C., Welch R.C., Kim H.J., George Jr. A.L., Sanders C.R. (2007). Preparation, functional characterization, and NMR studies of human KCNE1, a voltage-gated potassium channel accessory subunit associated with deafness and long QT syndrome. Biochemistry.

[B62-genes-01-00023] Gouas L., Nicaud V., Berthet M., Forhan A., Tiret L., Balkau B., Guicheney P., D.E.S.I.R. Study Group. (2005). Association of KCNQ1, KCNE1, KCNH2 and SCN5A polymorphisms with QTc interval length in a healthy population. Eur. J. Hum. Genet..

[B63-genes-01-00023] Akyol M., Jalilzadeh S., Sinner M.F., Perz S., Beckmann B.M., Gieger C., Illig T., Wichmann H.E., Meitinger T., Kääb S., Pfeufer A. (2007). The common non-synonymous variant G38S of the KCNE1-(minK)-gene is not associated to QT interval in Central European Caucasians: results from the KORA study. Eur. Heart J..

[B64-genes-01-00023] Marjamaa A., Newton-Cheh C., Porthan K., Reunanen A., Lahermo P., Väänänen H., Jula A., Karanko H., Swan H., Toivonen L., Nieminen M.S., Viitasalo M., Peltonen L., Oikarinen L., Palotie A., Kontula K., Salomaa V. (2009). Common candidate gene variants are associated with QT interval duration in the general population. J. Intern. Med..

